# Elucidating nanoscale mechanical properties of diabetic human adipose tissue using atomic force microscopy

**DOI:** 10.1038/s41598-020-77498-w

**Published:** 2020-11-24

**Authors:** J. K. Wenderott, Carmen G. Flesher, Nicki A. Baker, Christopher K. Neeley, Oliver A. Varban, Carey N. Lumeng, Lutfiyya N. Muhammad, Chen Yeh, Peter F. Green, Robert W. O’Rourke

**Affiliations:** 1 Department of Materials Science and Engineering, University of Michigan, Ann Arbor, MI 48109 USA; 2 Biointerfaces Institute, University of Michigan, Ann Arbor, MI 48109 USA; 3 Graduate Program in Immunology, University of Michigan, Ann Arbor, MI 48109 USA; 4 Graduate Program in Cellular and Molecular Biology, University of Michigan, Ann Arbor, MI 48109 USA; 5 Department of Surgery, Section of General Surgery, University of Michigan Medical School, 2210 Taubman Center-5343, 1500 E. Medical Center Drive, Ann Arbor, MI 48109-5343 USA; 6 Department of Pediatrics and Communicable Diseases, University of Michigan Medical School, Ann Arbor, MI 48109 USA; 7 Present Address: National Renewable Energy Laboratory, Golden, CO 80401 USA; 8 Department of Surgery, Veterans Affairs Ann Arbor Healthcare System, Ann Arbor, MI 48109 USA; 9 Division of Biostatistics, Department of Preventive Medicine, Feinberg School of Medicine, Northwestern University, Chicago, USA; 10 Present Address: Department of Materials Science and Engineering, Northwestern University, Evanston, IL 60201 USA

**Keywords:** Biophysics, Diseases

## Abstract

Obesity-related type 2 diabetes (DM) is a major public health concern. Adipose tissue metabolic dysfunction, including fibrosis, plays a central role in DM pathogenesis. Obesity is associated with changes in adipose tissue extracellular matrix (ECM), but the impact of these changes on adipose tissue mechanics and their role in metabolic disease is poorly defined. This study utilized atomic force microscopy (AFM) to quantify difference in elasticity between human DM and non-diabetic (NDM) visceral adipose tissue. The mean elastic modulus of DM adipose tissue was twice that of NDM adipose tissue (11.50 kPa vs. 4.48 kPa) to a 95% confidence level, with significant variability in elasticity of DM compared to NDM adipose tissue. Histologic and chemical measures of fibrosis revealed increased hydroxyproline content in DM adipose tissue, but no difference in Sirius Red staining between DM and NDM tissues. These findings support the hypothesis that fibrosis, evidenced by increased elastic modulus, is enhanced in DM adipose tissue, and suggest that measures of tissue mechanics may better resolve disease-specific differences in adipose tissue fibrosis compared with histologic measures. These data demonstrate the power of AFM nanoindentation to probe tissue mechanics, and delineate the impact of metabolic disease on the mechanical properties of adipose tissue.

## Introduction

Obesity-associated type 2 diabetes (DM) presents a distinct challenge to the nation’s health care system. In 2015, DM was the seventh leading cause of death in the US, and the Center for Disease Control projects DM prevalence rates may exceed 30% by 2050^1–4^. These data suggest a critical need for improved understanding of mechanisms underlying the pathogenesis of DM. Adipose tissue, especially intra-abdominal or visceral adipose tissue (VAT), relative to subcutaneous adipose tissue (SAT), plays a central role in DM pathogenesis. As adipose tissue mass expands beyond its nutrient storage capacity, adipocyte metabolic dysfunction ensues, leading to systemic nutrient overflow, nutrient toxicity in multiple non-adipose tissues, and ultimately, metabolic disease. Fibrosis is an important feature of the complex processes that lead to adipose tissue failure. As adipocytes undergo apoptosis in response to nutrient excess, an inflammatory and fibrotic response is directed towards dead and dying adipocytes, potentiating adipocyte metabolic dysfunction. Quantitative and qualitative changes in the adipose tissue extracellular matrix (ECM) are a dominant feature of murine and human obesity, but published data regarding the nature and directionality of these changes are conflicting, and their impact on adipose tissue function are not well defined. In addition, the majority of published literature reports histologic features and gene expression patterns in adipose tissue^5–10^. In contrast, literature addressing the effect of changes in the ECM on adipose tissue mechanics in the context of metabolic disease is sparse. This is an important point for investigation, given data demonstrating that cellular metabolism is influenced by exogenous mechanical forces in adipose and other tissues^11,12^.

Atomic force microscopy (AFM) nanoindentation measures tissue mechanics with nanoscale resolution by applying a small compressive force and extracting the Young’s, or elastic, modulus^13^. Only a few studies have applied AFM to cells derived from adipose tissue^14–19^, several of which attempted to directly measure mechanical properties, but none of these studies applied AFM to intact adipose tissue or investigated disease correlations. Related to this point, AFM has been utilized to investigate the mechanical properties of whole human tissues, including those from the brain^20^, eye^21^, and heart^22,23^, providing important insights for sample preparation and mechanical property analyses of our human adipose tissues. The goal of this work was to determine if AFM could define disease-specific mechanical characteristics of human adipose tissue, thus bridging an important knowledge gap regarding the effect of fibrosis on adipose tissue elasticity in the context of DM. We used AFM to determine elastic moduli in VAT from a cohort of obese humans with and without DM. We hypothesized that DM would be associated with increased tissue stiffness, *i.e.* higher elastic modulus.

## Results

### AFM nanoindentation of adipose tissue

**Figure 1:**
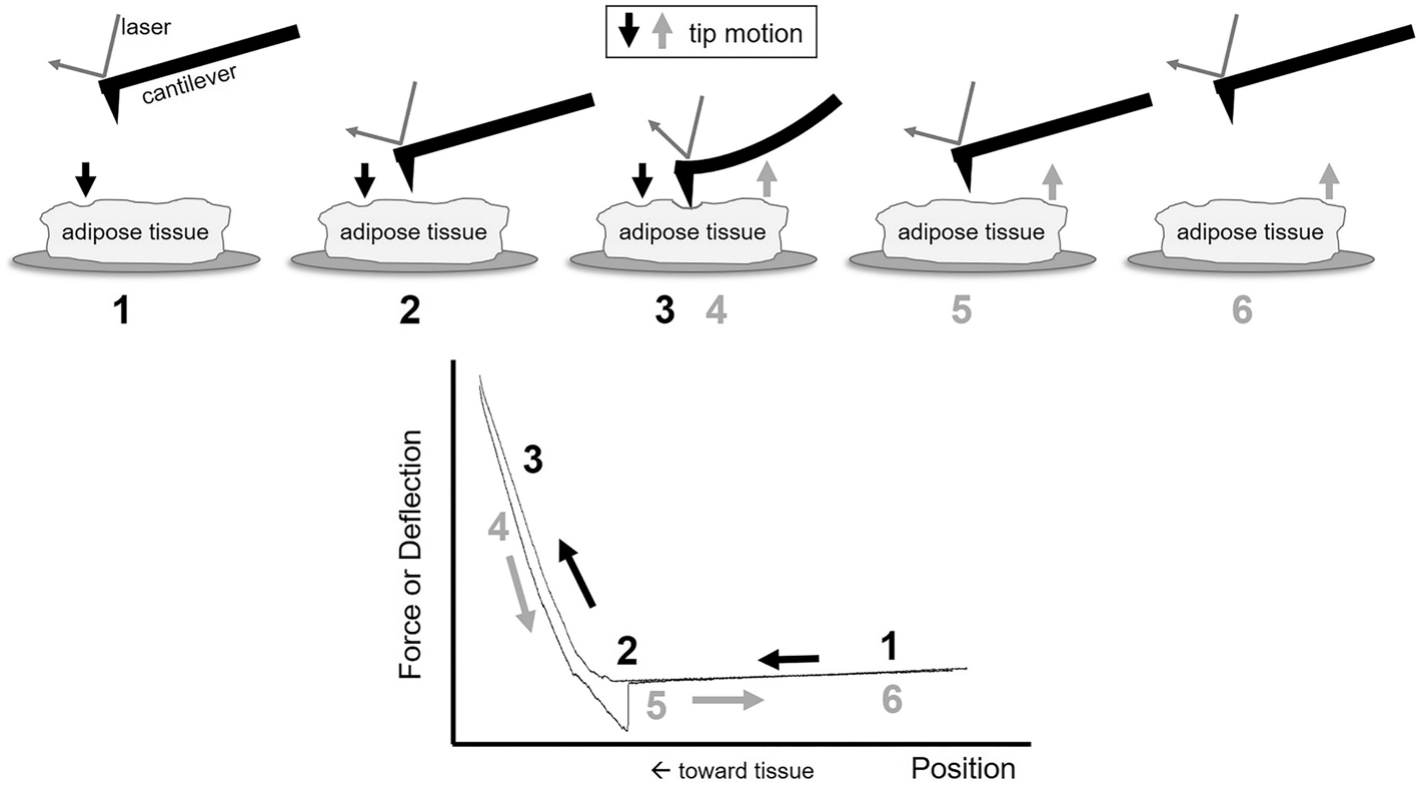
Schematic of AFM nanoindentation measurement with example raw force-indentation data. At point 1, the cantilever is removed from interaction with the tissue surface. The cantilever approaches the tissue surface (point 2), ultimately contacting and indenting into the tissue (points 3 and 4). Once the set point cantilever deflection has been reached (corresponding with a set force), the tip is retracted from the tissue surface (points 5 and 6). On the retraction curve, it is not uncommon to see a small spike in deflection as the tip pulls away from the sample (between points 4 and 5).

AFM nanoindentation was employed to study the mechanical properties of DM and NDM VAT from 17 obese humans (Fig. 1, Table 1). Mechanical properties encompass several individual features of materials, including but not limited to hardness, stiffness, elasticity, and strength. Here, we are primarily interested in elasticity, or the ability of the material to retain its original shape after a force is applied. Dependent on the applied force direction, however, different values, or moduli, of elasticity can be obtained. For example, if the force is in the shear direction, the shear modulus is measured; if the force is compressive or elongating in the linear direction, the Young’s modulus is measured. This linear compressive stress is distinct from uniform compression from which bulk modulus can be attained. In AFM nanoindentation, a small compressive force is applied to the sample via the AFM tip, and the Young’s, or elastic, modulus is extracted by fitting the linear portion of the force-distance curves.

**Table 1:** Subject demographics.

	AFM, adipocyte sizing, Sirius Red			Hydroxyproline		
	DM (n = 8)	NDM (n = 9)	*p* value*	DM (n = 12)	NDM (n = 15)	*p* value*
**Demographics**						
Age	45 (6.5)	44 (5.2)	0.676	46 (7.5)	42 (4.3)	0.156
BMI	45 (8.1)	42 (6.6)	0.453	45 (7.4)	48 (6.5)	0.283
HbA1c	6.3 (0.8)	5.2 (0.3)	0.003	6.7 (0.7)	5.3 (0.2)	< 0.001
**Comorbid diseases**						
Hypertension	50%	22%	0.335	58%	20%	0.057
Sleep apnea	50%	56%	> 0.999	58%	33%	0.258
Hyperlipidemia	25%	0%	0.206	42%	7%	0.060
**Medications**						
ACE inhibitor	25%	22%	> 0.999	17%	20%	> 0.999
β-Blocker	0%	0%	> 0.999	17%	0%	0.188
Statin	50%	0%	0.029	50%	7%	0.024
Insulin	25%	0%	0.206	42%	0%	0.010
Metformin	100%	0%	< 0.001	67%	13%	0.007
Sulfonylurea	12%	0%	0.471	8%	0%	0.444
Thiazolidine-dione	12%	0%	0.471	8%	0%	0.444

*Independent t-test and Fisher’s exact test were used to compare continuous and dichotomous variables respectively between DM and NDM groups; age (years), BMI (kg/m^2^), and HbA1c (%) shown as mean with standard deviation in parentheses.

**Figure 2:**
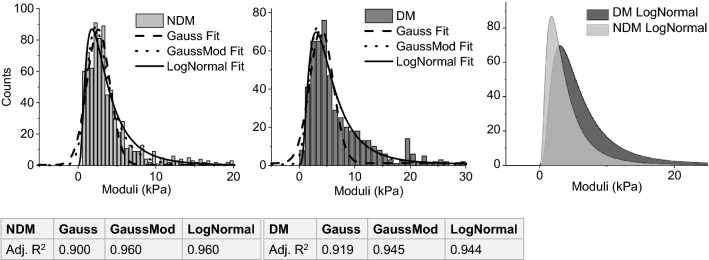
Raw DM and NDM moduli data with Gauss, GaussMod, and LogNormal distribution fits (left). LogNormal distribution fits shaded for both DM and NDM adipose tissues (right).

From all tissue samples, 764 NDM and 560 DM force-distance curves were analyzed to yield elastic moduli. The values of elastic moduli of both NDM and DM populations were binned and plotted as histograms, and Gaussian, exponentially modified Gaussian (GaussMod^24^), and log-normal (LogNormal^25^) distributions were applied to each histogram. As noted from fitting statistics, exponentially modified Gaussian and log-normal provided superior fits (R^2^ > 0.94) to the data, relative to the normal distribution fit (R^2^ ~ 0.9–0.91). The elastic moduli data are positively skewed, suggesting a poorer fit for the normal Gaussian distribution (Fig. 2). These “best” distribution fits of exponentially modified Gaussian and log-normal are consistent with previous reports of biomaterial and human brain tissue moduli extracted using AFM nanoindentation^20,26^. The positive-skewed nature of data distributions was further confirmed by investigating the normal (Gaussian), exponential, and log-normal probability plots (Figure S1), with NDM and DM patient data percentiles mapping most closely to the log-normal distribution (Fig. 2).

**Figure 3:**
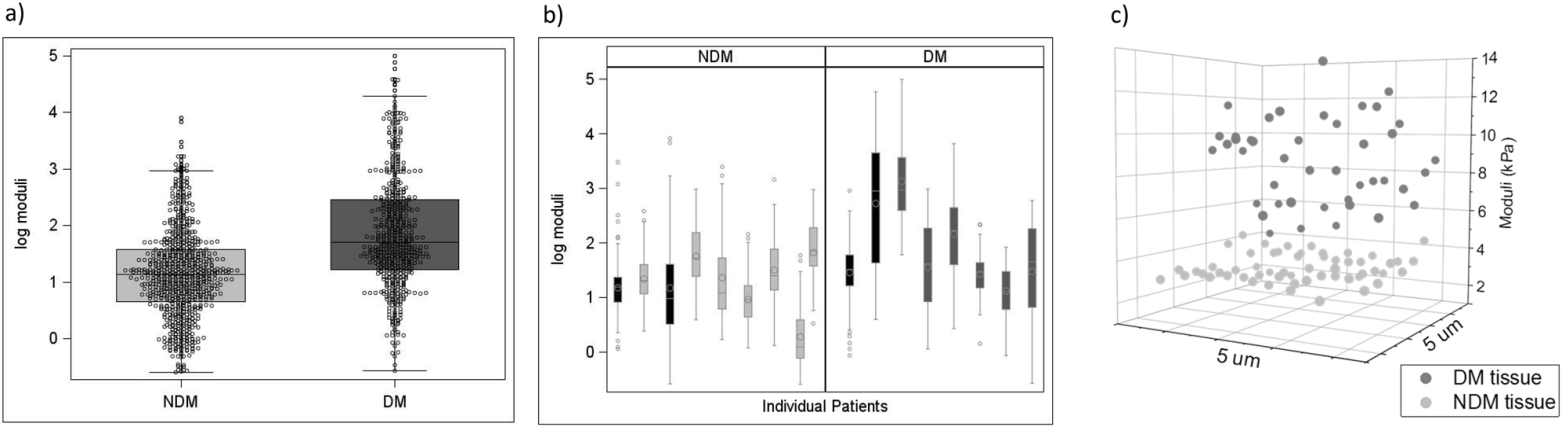
(**a**) Jitter plots of log-transformed elastic moduli of tissues from NDM and DM populations, center bar represents means, boxes represent interquartile ranges, and error bars represent standard deviations. (**b**) Box plots of log-transformed elastic moduli for individual subjects (subjects from each population that were measured twice are marked black) center circles represents means, boxes represent interquartile ranges, and error bars represent standard deviations. (**c**) Locoregional elastic moduli maps for two representative DM (dark grey dots) and NDM (light grey dots) 5 × 5 µm tissue samples. One dot represents a single elastic modulus value extracted from a 5 × 5 µm region of tissue.

Due to the skew of the data, a natural logarithmic transformation of the moduli data was performed prior to investigation of relevant statistics of DM and NDM adipose tissue moduli. Box plots for each population demonstrate that mean moduli of DM adipose tissue is greater than moduli of NDM adipose tissue (Fig. 3a).

### Disease-specific differences in adipose tissue nanoelasticity

Transformed log outcomes and outcomes of mean, standard deviation, and median of adipose tissue moduli data are shown in Table 2. The mean elastic moduli for the NDM and DM tissues were 4.48 ± 4.81 kPa and 11.50 ± 16.79 kPa, respectively. These statistics confirm that DM VAT, relative to NDM VAT, exhibits a larger elastic modulus with greater standard deviation than NDM VAT. The medians for NDM and DM elastic moduli are 3.12 kPa and 5.51 kPa, respectively. These medians are less than the calculated means for both populations, further confirming the right- or positive-skewed nature of the moduli data.

**Table 2:** Adipose tissue moduli statistics.

	NDM log outcome (kPa)	NDM outcome (kPa)	DM log outcome (kPa)	DM outcome (kPa)
N	764		560	
Mean	1.15	4.48	1.87	11.50
SD	0.79	4.81	1.00	16.79
Median	1.14	3.12	1.71	5.51

*N* number of data points measured, *SD* standard deviation.

With statistics suggesting NDM and DM moduli values are right-skewed and that NDM adipose tissue has a smaller average elastic modulus, determining the significance of the difference in elastic moduli between NDM and DM tissue is critical. To this end, a linear mixed model with random subject effect with the log moduli as the outcome and diabetic status as the predictor was employed. The model reveals that DM VAT has a larger elastic modulus than NDM VAT by 1.851 times kPa (average log modulus of 0.6156 times kPa, *p* value = 0.0488, 95% confidence interval 1.055–3.248) (Table S1).

### Locoregional variability of elasticity

The standard deviations and interquartile ranges of moduli data from DM and NDM populations extracted from statistical analyses indicate greater variability in DM relative to NDM tissues (Fig. 3a). Importantly, this variability in tissue elasticity at the population level is also observed within individual samples as displayed in individual patient box plots, which demonstrate that the variability in moduli for each patient differs, with VAT from DM patients having greater variability in elastic moduli than VAT from NDM patients (Fig. 3b). As expected from population statistics, spatial moduli of DM tissue are generally larger than those of NDM tissue. In addition, significantly greater intra-patient variability was observed in locoregional moduli values in DM relative to NDM tissues. In representative tissue maps, DM moduli vary ~ 9 kPa across the sample, while NDM moduli vary by ~ 3 kPa (Fig. 3c). These moduli maps demonstrate substantial locoregional spatial variability in DM tissue elasticity.

### Histologic VAT phenotype in DM

**Figure 4:**
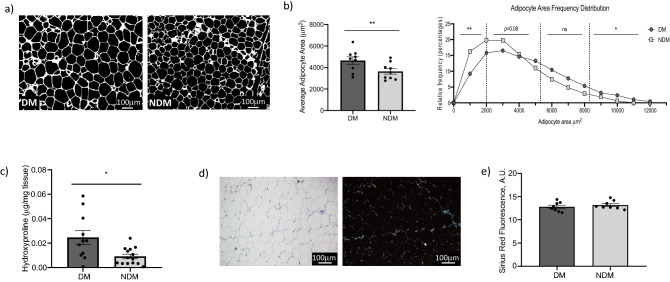
(**a**) Representative fluorescence images of sectioned VAT (scale bars: 100 µm) used for quantifying adipocyte area; (**b**) average adipocyte area (µm^2^) displayed as bar graphs (left, **p = 0.004 comparing DM and NDM average adipocyte area, controlling for age and BMI; error bars display standard error of mean), and adipocyte area frequency as histograms (right, *p* = 0.003 (**), 0.08, 0.184 (ns), 0.013 (*) respectively for each sizing group comparing DM and NDM adipocyte area frequency and controlling for age and BMI, generalized linear mixed model with negative binomial distribution); (**c**) hydroxyproline content in VAT: ordinate; hydroxyproline (µg/mg tissue); *p = 0.022 comparing DM and NDM groups, independent t-test; error bars display standard error of mean; (**d**) representative standard light microscopy (left) and polarized light (right) images of sectioned, Sirius Red-stained human VAT (scale bars: 100 µm); (**e**) quantified Sirius Red staining in VAT comparing DM and NDM groups; *p* = 0.437 comparing DM and NDM groups, independent t-test; error bars display standard error of mean.

To define histologic correlates of DM in VAT, we measured adipocyte size, a metric shown to correlate strongly with DM in human adipose tissue^8,27–32^, along with hydroxyproline content and Sirius Red staining as surrogates for tissue collagen content, in VAT sections. Adipocyte size and Sirius Red content were performed on VAT from the same 17 obese humans studied with AFM; hydroxyproline assay was performed on VAT from a separate cohort of 28 obese subjects (Table 1). Adipocyte size was increased in DM relative to NDM VAT (Fig. 4a,b).Hydroxyproline content was increased in DM relative to NDM VAT (Fig. 4c) and correlated with serum HbA1c (Figure S2). No difference was observed in Sirius Red staining in VAT between DM and NDM groups (Fig. 4d,e).

## Discussion

Our primary finding is that DM is associated with increased elastic moduli in human VAT, consistent with increased tissue stiffness. Few published studies measure mechanical properties at the nanoscale of human adipose tissue, and this is, to the best of our knowledge, the first report of decreased nanoscale tissue elasticity of human VAT in the context of metabolic disease. Current published literature provides conflicting results regarding adipose tissue ECM deposition and mechanics in metabolic disease, with some studies demonstrating increased ECM deposition and stiffness in DM and obesity via histology and macroscopic elastography measurements^33,34^, while others demonstrate the opposite^8^. These conflicting findings result in part from a wide range of methodologies used to study ECM and tissue mechanics. Furthermore, whether histologic measures of ‘fibrosis” and measurements of macroscale tissue mechanics correlate with tissue mechanical properties at the cellular level and the nanoscale remains unknown. Our report is novel in that it is the first of which we are aware that demonstrates increased adipose tissue stiffness at the cellular/nanoscale. Our findings have important implications for disease pathogenesis, as increased matrix stiffness has been shown to exert adverse effects on adipocyte metabolic function, including decreased fatty acid catabolism and increased expression of pro-fibrotic molecules, effects that would be expected to exacerbate adipose tissue dysfunction in the context of metabolic disease.

An important secondary finding from these data is the observation of significant variance in adipose tissue elasticity. This variance was observed among patient samples, and was increased in DM relative to NDM VAT, as evidenced by greater standard deviation of elastic moduli in the DM population relative to the NDM population. Furthermore, we observed significant spatial variance in VAT elasticity within individual patient tissue samples, at the nanoscale level through investigation of point moduli values extracted from small tissue areas and this variability was greater in DM relative to NDM tissues. Variable severity of disease and effects on tissue fibrosis may explain the increased elastic modulus variance in DM relative to NDM tissues among patients. The observation of significant intra-patient (i.e. within individual patients) locoregional spatial variability in tissue elastic moduli suggests that wide spatial sampling may be necessary to fully define disease-specific mechanical features of adipose tissue. Finally, while DM tissue has larger elastic moduli and greater variability than NDM tissue, some patients, independent of disease, have greater variability in their adipose tissue moduli than other patients. Comparison of tissues from patients that were repeatedly measured with those measured once demonstrated that variability in moduli for each patient is not strictly related to number of samples per patient measured. Thus, while it may be worthwhile to measure multiple adipose tissue samples for each patient, it is perhaps as critical to survey a wide set of patients from NDM and DM populations. This observation has implications for future studies of adipose tissue mechanics.

To correlate AFM findings with accepted histologic measures of adipose tissue fibrosis, we studied Sirius Red staining, a collagen-avid dye, and hydroxyproline content, a biochemical measure of collagen content, in adipose tissue. We also studied adipocyte size, a well-established correlate of DM in human obesity, as demonstrated by our group and others^8,27–32^. We observed an increase in collagen content measured by hydroxyproline content in DM relative to NDM tissues, but no such correlation with Sirius Red staining. Published data regarding the association of adipose tissue “fibrosis” and ECM content with metabolic disease are conflicting, with some studies demonstrating a positive correlation between metabolic disease and ECM content as measured by histology, primarily Sirius Red staining, while others demonstrate the opposite^5–9,33^. Even fewer published data describe the relationship between histologic measures of fibrosis and adipose tissue mechanics. Together, our findings confirm that histologic measures of adipose tissue fibrosis may be inaccurate correlates of disease and tissue mechanics, and that techniques such as AFM that directly measure mechanical properties of tissues may be more accurate measures of disease associations. Consistent with this hypothesis, a single prior study demonstrated increased subcutaneous adipose tissue stiffness using macroelastography measurements in DM relative to NDM humans, but no difference in immunohistochemical measures of fibrosis in the same patient cohort^33^.

Our study was restricted to women within a relatively narrow age and BMI range, to eliminate age, BMI, and sex as confounders. Nonetheless, this small patient cohort does not permit controlling for other potential confounders such as medications and other metabolic diseases, nor eliminate possible effects of age and BMI on the observed results. Larger studies will be required to determine the role of sex, age, depot, and other clinical variables on adipose tissue mechanics. We did not perform in vivo measures of systemic insulin resistance such as glucose tolerance testing due to resource limitations, but we have demonstrated that our methods of categorizing clinical DM using clinical diagnosis and HbA1c levels correlate strongly with molecular and cellular characteristics of adipose tissue^8,9,35–38^, suggesting that DM is the main driver of the AFM associations observed. Furthermore, we demonstrate that histologic measurement of adipocyte size, which has been shown by our group and others^8,27–32^ to strongly correlate with DM status, exhibits a similar correlation in this patient cohort, further supporting a link between DM status and select measures of tissue phenotype in our cohort. We restricted study to VAT due to limited access to SAT in our patient population, and because VAT is more strongly associated with metabolic disease, suggesting it as a clinically relevant target. This study was not designed to determine the role of changes in tissue mechanics in regulating adipose tissue cellular metabolic function, but prior data from our group and others demonstrate impaired adipocyte metabolism associated with DM^35–38^, and together with the present findings, suggest a role for tissue mechanics in regulating these functions. AFM measures compressive elasticity (i.e. Young’s modulus) and in this case, we did not attempt to extract other moduli (e.g. shear). These different aspects of tissue mechanics may have distinct effects on cell function and disease pathogenesis, and represent topics for future research. AFM nanoindentation is time-consuming and laborious, and unlikely to be applied to the clinical setting in the near future, but these data provide important insights into the effect of metabolic disease on adipose tissue mechanics and a launching point for further mechanistic studies of adipose tissue dysfunction.

This is the first study to demonstrate nanoscale level correlations of adipose tissue elasticity with metabolic disease in humans. We confirm increased elastic moduli, i.e. increased stiffness, of VAT in DM humans, corroborating macroscopic elastography data and providing novel insights into the role of metabolic disease in regulating adipose tissue mechanical properties at the nanoscale. Additionally, greater variability in elasticity was noted in DM relative to NDM VAT. These findings together identify altered tissue mechanics as a feature of adipose tissue dysfunction in metabolic disease.

## Methods

### Human subjects

Human subjects were consented with written consent and enrolled with approval from Institutional Review Boards at the University of Michigan and Veterans Affairs Ann Arbor Healthcare System. Enrollment was restricted to women to avoid sex as a confounder. Visceral adipose tissue (VAT) from the greater omentum and subcutaneous adipose tissue (SAT) from the abdominal wall was collected from 17 obese (body mass index (BMI) > 35 kg/m^2^) subjects during bariatric surgery. Diabetic (DM) subjects were defined by clinical diagnosis requiring treatment with medication and hemoglobinA1c (HbA1c)  ≥ 6.5%. Non-diabetic (NDM) subjects were defined by no clinical history of diabetes and hemoglobinA1c (HbA1c) < 5.7% per American Diabetes Association criteria^39^ (Table 1).

### Nanoindentation measurements

VAT was snap frozen in liquid nitrogen then cryo-cut into 10 µm sections onto poly-lysine-coated coverslips. Edges of coverslips were carefully glued to cell culture plates. After glue-drying, PBS was added to cover the samples, which were then placed in an Asylum AFM MFP3D chamber (Oxford Instruments PLC, Oxfordshire, UK). The AFM cantilever (Hydra-6-200N, AppNano, k ~ 0.035 N/m, f ~ 17 kHz) was lowered into the buffer solution of the sample, equilibrated for at least 2 h to allow the tip deflection to reach a stable value, then the tip spring constant was calibrated by thermal tuning in the solution, and the deflection invOLS (providing a ratio of nm to V) was measured by performing at least ten indentations on the plain cover slide, which was treated as a hard, incompressible surface. Several force maps of size 5 × 5 µm containing 64 evenly spaced indentation points were taken across the tissue surface to account for sample variability. During the nanoindentation measurement, the cantilever begins at a position removed from any interaction with the sample surface, and a laser targeting the back of the AFM tip records the cantilever deflection, and thus tip position. The cantilever is lowered until it contacts the sample surface, at which point a significant deflection change is recorded. The tip indents into the sample to a chosen deflection set point, which corresponds with a chosen force set point. Typical indentation forces were on the order of ~ nN.

### Force-distance curve analysis

The force-distance curves were analyzed using software provided by Asylum Research (Oxford Instruments PLC, Oxfordshire, UK) based on the Hertz model^40^. Other models, such as the Johnson-Kendall-Robert (JKR)^41^ and Derjaguin-Muller-Toporov (DMT)^42^ employed to understand polymer film mechanics^43^ were considered, but the Hertz model contains several assumptions that merit its use in this work, including small indentation forces within the linear elastic regime and negligible sample/tip adhesion. Use of a spherical tip geometry in the Hertz model was informed by the Asylum Research software that reports power law slope, with $F \propto {\delta ^{3/2}}$indicative of a spherical tip geometry. More information regarding this power law relationship is discussed in “Supplementary Information”. Assessment of power law with indentation depth is important for proper choice of model and is documented in other nanoindentation studies of human tissues^20–22^, and the 3/2 power law relationship in our case is shown in Figure S2. The calibrated tip information (invOLS and spring constant), tip material (Si, ν_Si_ ~ 0.27) and geometry (sphere with radius of 10 nm), and sample information (ν_adiposetissue_ ~ 0.5)^44^ were input into the model. The AFM tip radius was informed by company (Applied NanoStructures, Inc, Mountain View, CA, USA) specifications and not by direct measurement by the authors. Impact of the changing of tip radius on Young's modulus is discussed in “Supplementary Information”. The fitting was varied to include only the elastic, or linear, portion of indentation at depths corresponding to ~ 15% or less of the applied force. An example of an experimental curve with fitting is shown in Figure S2.

### Adipocyte area

Adipocyte area (µm^2^) was measured as described^8^ using fixed hematoxylin/eosin-stained sections imaged on an Olympus IX-81 fluorescent microscope using Texas Red channel (595-605 nM). Images were captured as multiple TIFF-gray-scale images and analyzed with Fiji image processing package, distribution of ImageJ (National Institutes of Health, Bethesda, MD, USA and Laboratory for Optical and Computational Instrumentation, University of Wisconsin, Madison, WI, USA). Adipocyte area was measured in 200–500 cells from multiple images per slide.

### Hydroxyproline assay

For tissue preparation, 100µL of ultrapure water was added to 10 mg of tissue and thoroughly homogenized using a Bead Bug homogenizer (Benchmark Scientific Inc., Sayreville, NJ, USA, Model D1030) and stainless steel beads, then transferred to a pressure-tight, screw-capped polypropylene vial and 100 µL of 10 M concentrated NaOH was added. Tubes were heated at 120 ° for 1 h. After alkaline hydrolysis, tubes were placed on ice and allowed to cool briefly before adding 100 µL of 10 M HCl to neutralize samples. Tubes were vortexed and centrifuged at 10,000×*g* for 5 min to separate lipid and insoluble debris. Cleared supernatants were transferred to fresh tubes and 10 µL of supernatant was used for hydroxyproline assay, performed utilizing manufacturer protocol (Sigma-Aldrich Inc., St Louis, MO, USA, Cat# MAK357).

### Sirius Red staining

Samples were sectioned with a Leica RM2155 Microtome (Leica Microsystems GmbH, Wezlar, Gemany) at 4 microns, then deparaffinized with a xylene wash, ethanol wash, and deionized water wash. Slides were then stained with enough Sirius Red/Fast Green Collagen stain (Chondrex Inc., Redmond, WA, USA, Cat #9046) to immerse the tissue section and stained for 30 min while covered to prevent evaporation. The dye was then carefully aspirated and slides were rinsed in deionized water until runoff was clear. Slides were then imaged using brightfield and polarized light microscopy with a Nikon E800 upright microscope. Polarized light images were analyzed for intensity using Fiji image processing package, distribution of ImageJ (National Institutes of Health, Bethesda, MD, USA and Laboratory for Optical and Computational Instrumentation, University of Wisconsin, Madison, WI, USA).

### Statistical methods

Characteristics of the study participants are summarized as descriptive statistics (Table 1). Every study participant has multiple outcome observations. Two DM and two NDM participants were measured twice. Due to repeated measurements of the outcome within the same study participant, a linear mixed model. was fitted to compare average elastic moduli between DM and NDM groups (Table S1). A linear mixed model captures the variability of measurements between individuals and the variability within measurements from an individual by including fixed and random effects^45^. Specifically, DM status was a fixed effect and each participant was a random effect in the linear mixed model. Because residuals of the linear mixed model were not normally distributed with equal variance, we used a natural logarithm transformation of the outcome and performed the linear mixed model with the same analysis strategy. The distribution of the natural logarithm transformation of the outcome is illustrated in box plots irrespective of multiple measurements. Mean elastic moduli and standard deviations for each group are reported on the raw and natural logarithm scales (Table 2).

For hydroxyproline and Sirius Red data, datasets were tested for outliers and outliers removed, then tested for normality and independent t-test used to compare DM and NDM groups. For adipocyte size data, analysis was done using SPSS Statistics (IBM SPSS Statistics for Mac, Version 27.0. Armonk, NY: IBM Corp.). The mean adipocyte area for each image was calculated. A linear mixed model was run with average adipocyte area as the output; DM status, age, and BMI as fixed effects; and patient as a random effect. Analysis of adipocyte area frequency was done using a generalized linear mixed model that accommodates a negative binomial distribution used for count data. Sizing groups were determined based on the overall size distribution and corresponding size percentiles. The frequency within each sizing group for each patient was counted. A generalized linear mixed model was run for each sizing group with DM status, age, and BMI as fixed effects; patient as a random effect; and total number of cells per patient as the offset.

### Ethical approval

All methods were carried out in accordance with relevant guidelines and regulations as outlined in the Springer Nature website. Human subjects were consented with written consent and enrolled with approval from Institutional Review Boards at the University of Michigan and Veterans Affairs Ann Arbor Healthcare System. Enrollment, consent, and all aspects of human subjects research were carried out in accordance with the Belmont Report from the National Research Act of 1974, and he Declaration of Helsinki set forth by the World Medical Association. This manuscript contains no human participants' names or other HIPAA identifiers.

## Supplementary information

Supplementary Information.

## Data Availability

All data generated and analyzed during the current study are included in the published article and its supplementary information files. All reagents will be freely provided upon reasonable request, except for human tissue and cell samples, and human subject clinical information or identifying information, which are not permitted to be shared due to IRB, HIPAA, and confidentiality constraints.

## Abbreviations

AFM: Atomic force microscopy
BMI: Body mass index
DM: Type 2 diabetes/diabetic
ECM: Extracellular matrix
HbA1c: Hemoglobin A1c
NDM: Non-diabetic
SAT: Subcutaneous adipose tissue
VAT: Visceral adipose tissue
