# Supplementary material for: Elucidating nanoscale mechanical properties of diabetic human adipose tissue using atomic force microscopy
Source: Sci Rep. 2020 Nov 24;10:20423. doi: 10.1038/s41598-020-77498-w (PMC7686328; doi:10.1038/s41598-020-77498-w)
Supplement: Supplementary file 1 — Supplementary Information. [file 41598_2020_77498_MOESM1_ESM.docx]

**Supplementary Information**

**Elucidating nanoscale mechanical properties of diabetic human adipose tissue using atomic force microscopy**

J. K. Wenderott^1,2,9^, Carmen G. Flesher^5^, Nicki A. Baker^5^, Christopher K. Neeley^5^, Oliver A. Varban^5^, Carey N. Lumeng^3,4,6^, Lutfiyya N. Muhammad^10^, Chen Yeh^10^, Peter F. Green^1,2,7,*^_,_ Robert W. O’Rourke^5,8,*^

^1^Department of Materials Science and Engineering, ^2^Biointerfaces Institute, ^3^Graduate Program in Immunology, and ^4^Graduate Program in Cellular and Molecular Biology, University of Michigan, Ann Arbor, MI 48109, USA; ^5^Department of Surgery, ^6^Department of Pediatrics and Communicable Diseases, University of Michigan Medical School, Ann Arbor, MI 48109, USA; ^7^Current address: National Renewable Energy Laboratory, Golden, CO 80401, USA; ^8^Department of Surgery, Veterans Affairs Ann Arbor Healthcare System, Ann Arbor, MI 48109, USA; ^9^Current address: Department of Materials Science and Engineering, Northwestern University, Evanston, IL 60201, USA; ^10^Department of Preventive Medicine, Division of Biostatistics, Feinberg School of Medicine, Northwestern University, Chicago, USA

^*^Corresponding Authors: Peter F. Green, Ph.D., National Renewable Energy Laboratory, Golden, CO 80401, USA, Office: (303)-275-3008; Fax: (303)-275-3097; Peter.Green@nrel.gov; Robert W. O’Rourke, M.D., University of Michigan, Department of Surgery, Section of General Surgery, 2210 Taubman Center-5343, 1500 E. Medical Center Drive, Ann Arbor, MI 48109-5343; Phone: (734) 647-9024; Fax: (734) 232-6188; rorourke@umich.edu

**Figure S1**. Probability plots for normal (left), exponential (center), and log-normal (right) distributions with percentiles for DM (top row) and NDM (bottom row) tissue moduli. The dark grey lines on the probability plots correspond to the reference lines and indicate the line on which the percentiles of the patient data (light grey circles) should fall if corresponding with a distribution (black lines are upper and lower percentiles). The datasets appear to match best with the log-normal distributions.


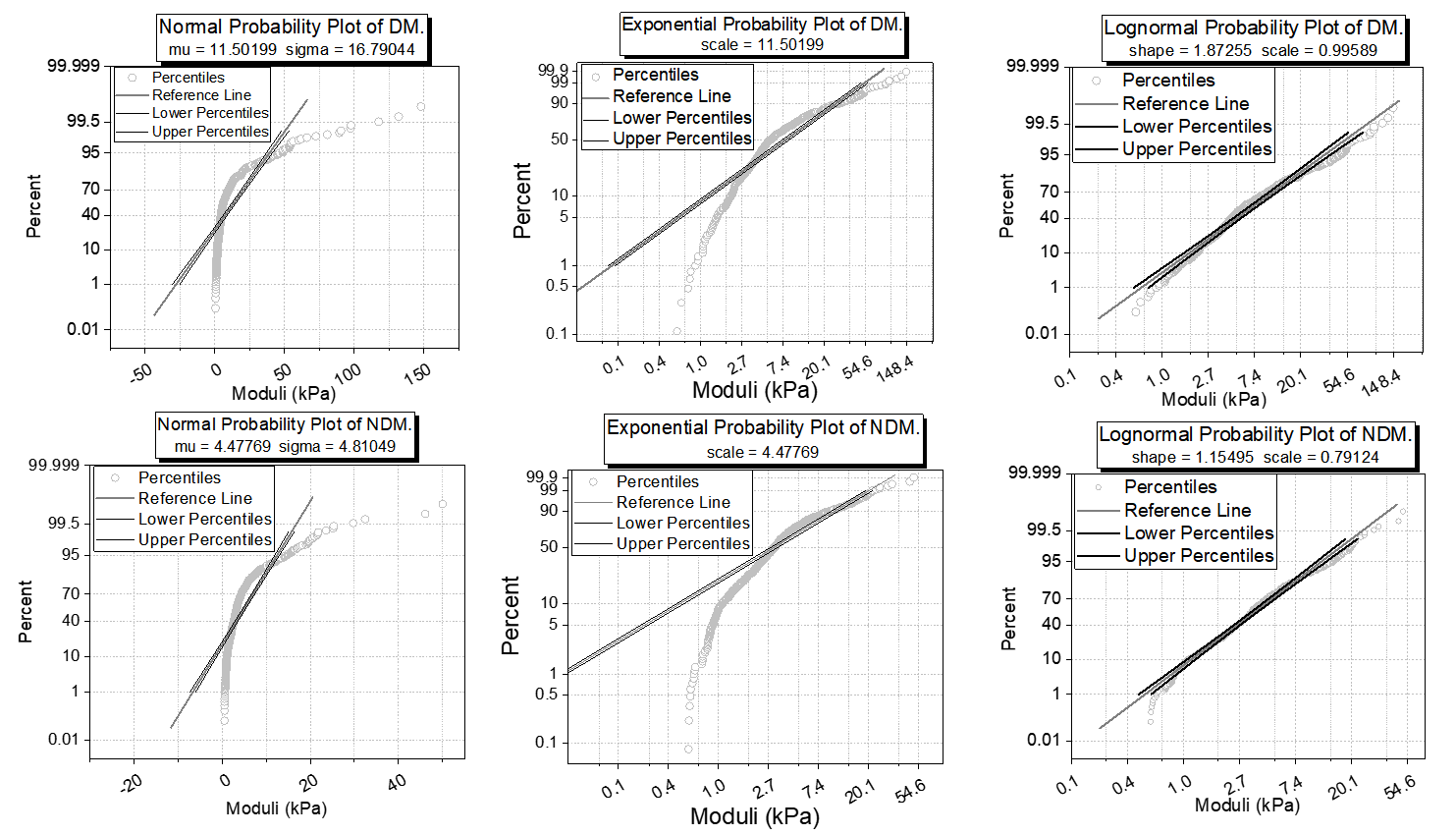


| **Effect** | **Estimate** | **Standard Error** | **DF** | **t Value** | **Pr ˃ \|t\|** |
| --- | --- | --- | --- | --- | --- |
|  |  |  |  |  |  |
| **Intercept** | 1.2630 | 0.1965 | 14.8 | 6.43 | ˂0.0001 |
| **DM** | 0.6156 | 0.2869 | 14.9 | 2.15 | 0.0488 |

**Table S1**. Estimates of linear mixed model that included a random intercept effect with log modulus as outcome and DM status as predictor. NDM is used as the reference group against which DM is compared.


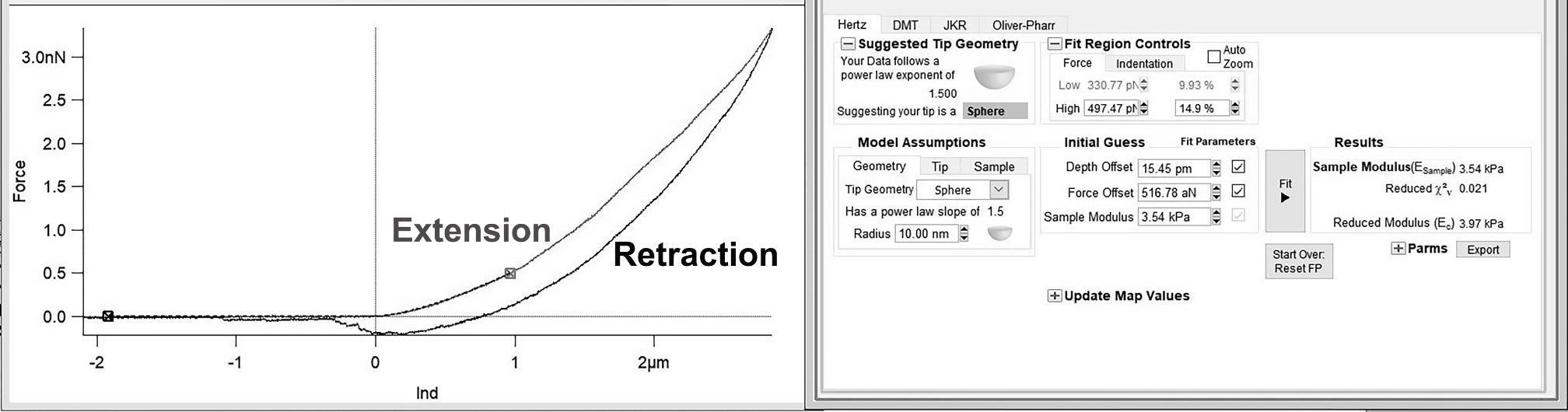


**Figure S2**. Example of experimental force-indentation curve of adipose tissue (top) and Asylum Research data fitting panel (bottom). With a fitting out to 15% of force applied, the power law exponent is 3/2, corresponding to spherical tip geometry. This fitting of percentage of force around 15% is typical for our data. Software information: Asylum Research MFP3D version 14.30.157; Igor Pro version 6.37; https://afm.oxinst.com/.


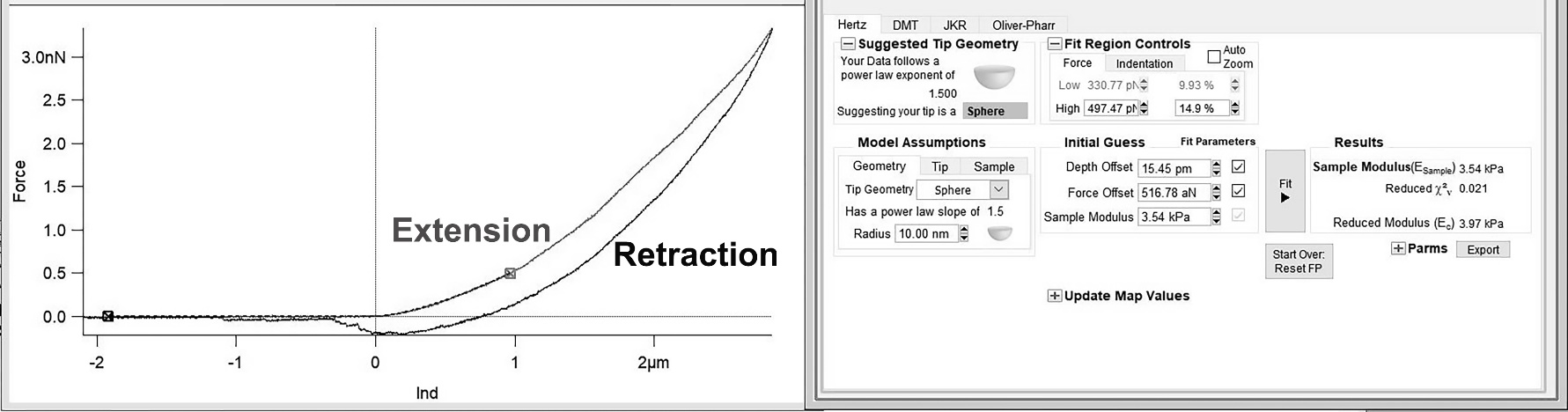


*Supplementary Information: Hertz Power Law Relationship Discussion*

In the sphere-sphere contact problem, the Hertz theory of contact of two bodes yields:

$E= \frac{3}{4}\left( 1-\nu^{2} \right)P{(\frac{1}{R^{*}}+\frac{1}{R})}^{\frac{1}{2}}h^{-\frac{3}{2}}$ *.* (1)

where *E* is the Young's modulus of the sample, *ν* is the Poisson's ratio of the sample, *P* is the applied load, *R^*^* is the indenter's radius, *R* is the sample's radius, and *h* is the indentation depth. If *R* at least several times larger than *R^*^*, the sample is large (*R**~10 nm, nominal specifications provided by the company AppNano^1^ is many times smaller than the cryo-cut tissue sample radii so *R>>R**) the equation becomes:

$E= \frac{3}{4}\left( 1-\nu^{2} \right)P{{(R}^{*})}^{-\frac{1}{2}}h^{-\frac{3}{2}}$ *.* (2)

and

$P= \frac{4}{3}\frac{E}{\left( 1-\nu^{2} \right)}{{(R}^{*})}^{\frac{1}{2}}h^{\frac{3}{2}}$ *.* (3)

This equation (3) reveals that *P* (or *F*) $\propto$ *h^3/2^* , the power law relationship used in our analysis.

In the unlikely event that the tip radius was an order of magnitude larger than the *R**=10 nm used in our analysis, then based on equation 2, the actual modulus would be a factor of 3 smaller. It is important to note that we used numerous tips in numerous experiments and received consistent results (*e.g.*, power law relationship in force distance curves) for the samples. Finally, and most important, the observed Young's moduli difference between the two tissue populations would still not be impacted by differences between the tip radii.

**References**

1. Silicon nitride cantilevers, SiN probes, Hydra, Nitra Tall. *Appnano* at <http://www.appnano.com/://www.appnano.com/>
